# Dynamic Deformation Behavior and Fracture Characteristics of a near α TA31 Titanium Alloy at High Strain Rates

**DOI:** 10.3390/ma15217599

**Published:** 2022-10-29

**Authors:** Weixin Yu, Xiaofen Li, Jinyong Zhang, Shusen Hou, Yifan Lv

**Affiliations:** 1 School of Mechanical and Electrical Engineering, Xinxiang University, Xinxiang 453003, China; 2 School of Materials and Physics, China University of Mining and Technology, Xuzhou 221116, China; 3 Luoyang Ship Material Research Institute, Luoyang 471000, China

**Keywords:** TA31 titanium alloy, dynamic impact compression, deformation behavior, microstructure evolution, fracture characteristics

## Abstract

The quasi-static and dynamic impact compression tests of the TA31 titanium alloy were conducted at the strain rates from 0.001 s^−1^ to 4000 s^−1^ and deformation temperatures from 293 K to 773 K, and the TA31 titanium alloy showed typical elastic-plastic characteristics. In the initial stage of compression (elastic deformation), the stress and strain are proportional, and the stress–strain curve is a straight line. In the plastic deformation stage, the flow stress decreases significantly with the increase of deformation temperature, while the strain rate has no significant effect on the flow stress during dynamic compression. A constitutive model has been established to predict the flow stress, and the relative error is 2.32%. It is shown by observing the microstructure that when the deformation temperature is 293 °C, and the strain rate reaches 1600 s^−1^, a shear band with an angle of about 45° to the axial direction of the specimen appears, and the severe shear deformation makes the α phase in the shear band fibrous and contains high-density dislocations. The formation process of the shear band and its influence on fracture are analyzed and discussed.

## 1. Introduction

TA31 is a near α titanium alloy, and its nominal composition is Ti-6Al-2Zr-1Mo-3Nb. TA31 titanium alloy has good weldability and plastic toughness in addition to the common excellent properties of titanium alloys, making it an ideal titanium alloy material for ships. In the application process of these fields, titanium alloy as a structural material may bear complex dynamic loading. The practice shows that under the impact load, the mechanical behavior of the material is quite different from that under quasi-static and low strain rates conditions [1,2,3,4,5], and understanding the mechanical response and fracture characteristics under dynamic load is very important to prevent accidental engineering failure of structural components.

There have been some reports on the dynamic response and deformation mechanism of titanium alloys under dynamic shock loading. Liu et al. [6] studied the dynamic compressive properties of Ti-6Al-4V alloy by high-speed impact test and found that the flow stress showed obvious strain rate and temperature sensitivity; that is, it increased with the increase of strain rate and the decrease of temperature. Li et al. [7] measured the dynamic mechanical properties of Ti17 titanium alloy using a split Hopkinson compression bar system, and the experimental results show that the flow stress is higher than the static values, and the failure is sensitive to strain rate. The mechanical responses of the five titanium alloys were characterized, and it was found that the strain hardening effect of the titanium alloys was very pronounced at low strain rates, but it might be reduced by the presence of a β-phase stabilizer at high strain rates [8]. Zhu et al. [9] investigated the mechanical response and microstructure evolution of pure titanium under quasi-static and dynamic loading and found that dynamic compression exhibited higher yield stress and peak stress than quasi-static compression. Microstructural observations revealed that shear bands composed of ultrafine equiaxed grains appeared in the dynamic specimens, which were believed to be the result of subgrain rotational dynamic recrystallization. Shi et al. [10] predicted the flow stress of a near α Ti-8Al-1Mo-1V titanium alloy during dynamic compression by the J-C model and found that an adiabatic shear band began to form at the strain rate of 2100 s^−1^. Ran et al. [11] found that the Ti-5Al-5Mo-5V-1Cr-1Fe alloy undergoes shear fracture under shock loading, and the failure occurs along a plane at an angle of about 45° to the compression axis. The microstructure also has a certain influence on the dynamic behavior of titanium alloy. Arab et al. [12] studied the dynamic impact deformation behavior and microstructure transformation of TA15 titanium alloy under different heat treatment states and found that the strain rates required for shear band formation under different microstructure states are different. The existing literature shows that titanium alloy is prone to a shear deformation band caused by material flow instability under high-speed impact conditions, and the deformation mechanism inside the shear band is studied, including twinning, dislocation slip, and recrystallization. However, there are few reports on the number of shear bands and the mechanism of shear band broadening during the dynamic impact of titanium alloys.

In this paper, the experimental technique of split Hopkinson pressure bar was applied to TA31 titanium alloy, and the deformation behavior of TA31 titanium alloy under high-speed impact loading was analyzed and modeled, especially the microstructure evolution and fracture behavior of TA31 titanium alloy during impact deformation were studied.

## 2. Materials and Methods

**Figure 1:**
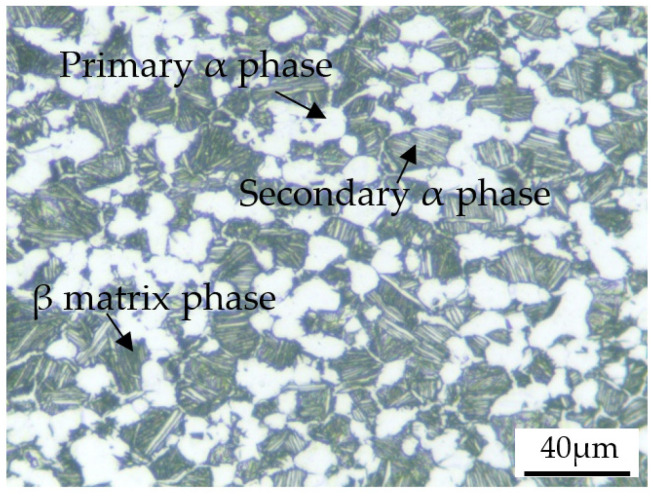
Microstructure of the TA31 titanium alloy in the as-received condition.

The TA31 titanium alloy used in this study was a bar with a diameter of 25 mm. The manufacturing process of the bar was as follows: first, the ingot was prepared by three times of vacuum consumable melting, then the ingot was heated to the β-phase field and forged, and the final forging temperature was in the α + β phase field. The chemical composition of the TA31 titanium alloy is given in Table 1. The α + β → β phase transition temperature determined by differential thermal analysis is about 1000 °C. The optical microstructure of the TA31 titanium alloy in the as-received condition is shown in Figure 1, which consists of equiaxed primary α phase with a volume fraction of about 50%, lamellar secondary α phase with a volume fraction of about 35%, and β matrix phase. The specimens used in the quasi-static compression tests were 8 mm in diameter and 12 mm in height, and the specimens with a diameter of 5 mm and a height of 4 mm were used in the dynamic impact tests. All the specimens were machined from the TA31 titanium alloy bar.

The quasi-static compression tests were performed at a room temperature of 293 K and strain rate of 0.001 s^−1^ on an electro-hydraulic servo universal testing machine MTS-370-250 KN. The dynamic impact tests were performed using a split Hopkinson pressure bar apparatus. Because the service temperature of TA31 titanium alloy components generally does not exceed 773 K and the maximum speed that the equipment can reach is 4000 s^−1^, the strain rates of 700 s^−1^, 1600 s^−1^, 2800 s^−1^, and 4000 s^−1^ and temperatures of 293 K, 373 K, 573 K, and 773 K were selected for the dynamic impact tests. The technical method and principle of the split Hopkinson pressure bar experiment are the same as those in reference [13].

After compression, the compressed specimens were cut along the axial direction by wire cutting, and the cut surfaces were prepared into a metallographic sample. The metallographic samples were ground, polished, and etched for microstructure observation. The ratio of etchant was HF:HNO_3_:H_2_O_2_:H_2_O = 1:2:7:10. Optical microstructure observation and photographing were carried out on Leica DMI 5000 M microscope. The compressed specimens were sliced along the axial direction with a slice thickness of 0.5 mm, and then ion-thinned to prepare a transmission electron microscope sample. A transmission electron microscope experiment was carried out on CM200. A VMH-104 microhardness tester was used to test the microhardness of different regions of the compressed specimens under the load of 200 N and the holding time of 10 s.

## 3. Results

### 3.1. Mechanical Response

**Figure 2:**
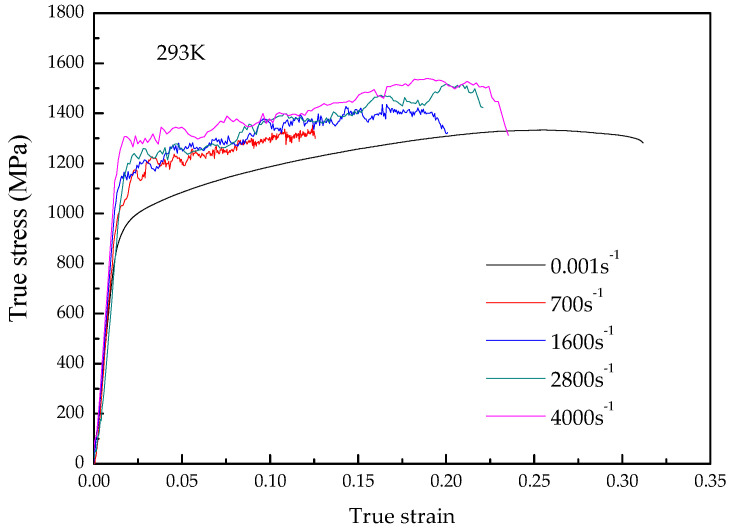
True stress–strain curves of TA31 alloy compressed at a temperature of 293 K and strain rates of 700 s^−1^, 1600 s^−1^, 2800 s^−1^, and 4000 s^−1^.

**Figure 3:**
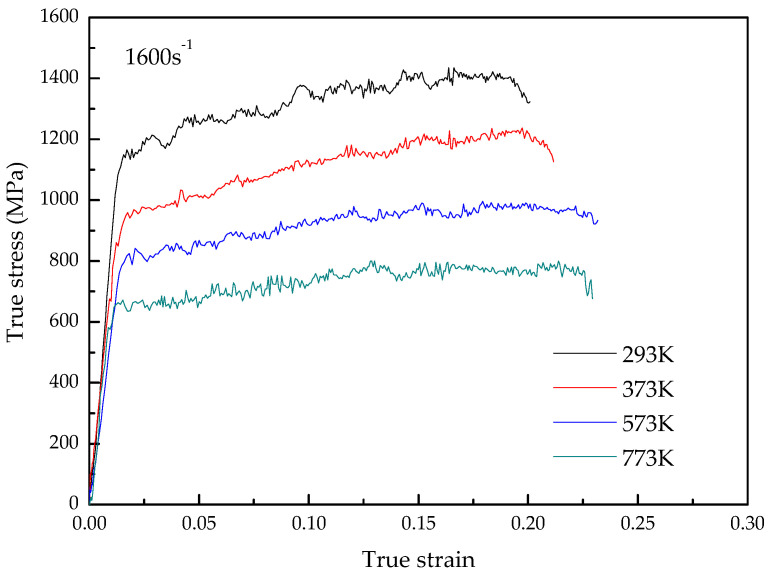
True stress–strain curves of TA31 alloy compressed at strain rates 1600 s^−1^ and temperatures of 293 K, 373 K, 573 K, and 773 K.

The stress–strain curves of TA31 titanium alloy compressed at strain rates ranging from 0.001 s^−1^ to 4000 s^−1^ and temperatures ranging from 293 K to 773 K are shown in Figure 2 and Figure 3, respectively.

It can be seen from Figure 2 and Figure 3 that the TA31 titanium alloy shows typical elastic-plastic characteristics. In the elastic deformation stage, the flow stress increment is proportional to the strain increment, and the stress–strain curve is a straight line, the slope of which is the elastic modulus of the TA31 titanium alloy. After the straight line ends, the deformation enters the plastic deformation stage. In the plastic deformation stage, the flow stress increases gradually with the increase of strain, indicating that the material exhibits an obvious work-hardening effect.

The deformation temperature has a significant effect on the flow stress, and the flow stress decreases with the increase in temperature, which indicates that the dynamic impact deformation of the TA31 titanium alloy is a thermal activation process. Compared with the deformation temperature, the effect of strain rate on flow stress during dynamic compression is insignificant.

### 3.2. Constitutive Model

The J-C model can accurately describe the deformation behavior of metal materials in the process of dynamic impact deformation, and its form is

(1) $$\sigma =(A+B\varepsilon^{n})(1+C\ln{\dot{\varepsilon }}^{*})(1-T^{*}{}^{m})$$

where *σ* and *ε* are flow stress and strain, respectively, ${\dot{\varepsilon }}^{*}$ represent $\dot{\varepsilon }/{\dot{\varepsilon }}_{0}$ in which $\dot{\varepsilon }$ and ${\dot{\varepsilon }}_{0}$ is strain rate and the reference strain rate (0.001 s^−1^) respectively, T * represents (*T* − *T_r_*)/(*T_m_* − *T_r_*) in which *T*, *T_m_,* and *T_r_* is the deformation temperature, the melting temperature (about 1923 K) and the room temperature (293 K) respectively, *m* and *n* represent thermal softening exponent and strain hardening exponent respectively, *A* represent yield stress at *T_r_* and ${\dot{\varepsilon }}_{0}$, *B* and *C* is strain hardening coefficient and strain rate hardening coefficient respectively [13,14,15].

The value of A can be determined to be 893 MPa by a quasi-static compression test.

At 293 K and 0.001 s^−1^, Equation (1) is

(2) $$\sigma =A+B\varepsilon^{n}$$

Equation (2) is equivalently transformed into

(3) ln(σ−A)=lnB+nlnε

**Figure 4:**
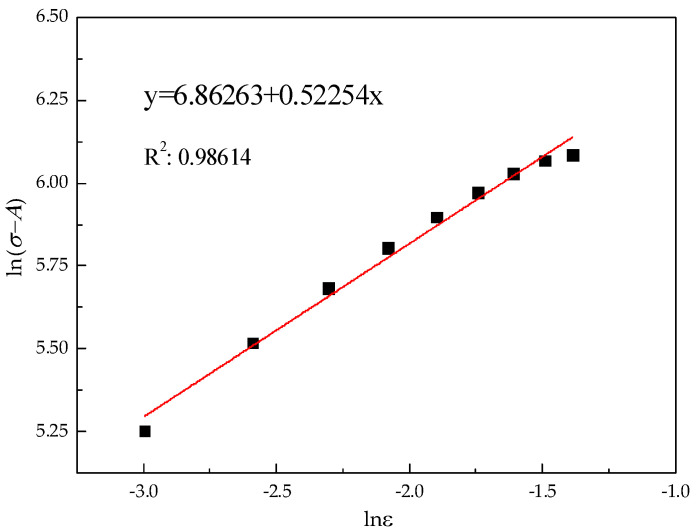
The ln(σ−A) vs. lnε linear fitting line.

The value of *B* and *n* obtained from Figure 4 are 956 and 0.523, respectively.

**Figure 5:**
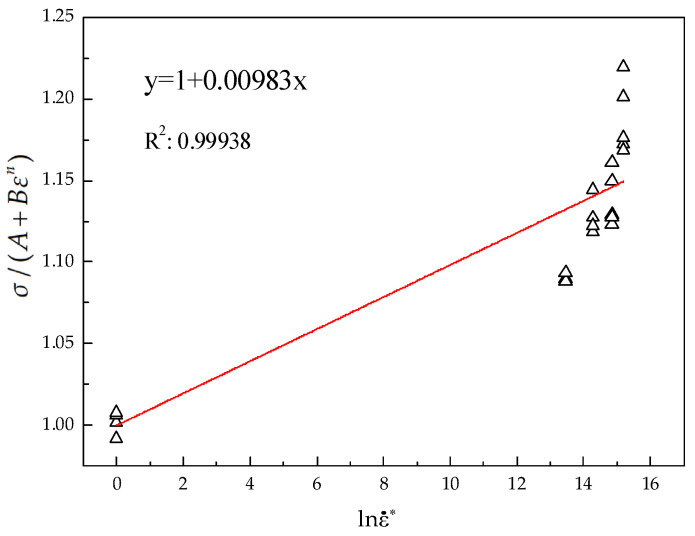
The $\sigma /(A+B\varepsilon^{n})$ vs. $\ln{\dot{\varepsilon }}^{*}$ linear fitting line.

At 293 K, Equation (1) is simplified to

(4) $$\sigma /(A+B\varepsilon^{n})=1+C\ln{\dot{\varepsilon }}^{*}$$

where *C* is the slope of the linear fitting line in Figure 5, which is 0.0098.

**Figure 6:**
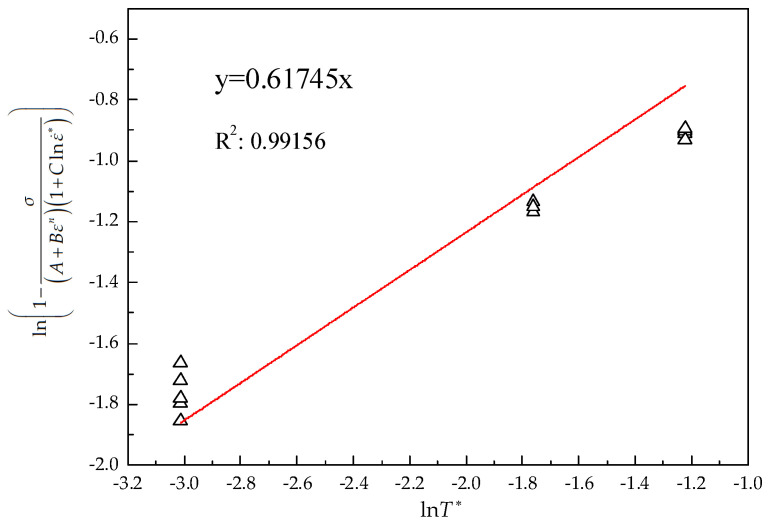
The $\ln\left(1-\sigma /\left(\left(A+B\varepsilon^{n}\right)\left(1+C\ln{\dot{\varepsilon }}^{*}\right)\right)\right)$ vs. $\ln T^{*}$ linear fitting line.

Transform the form of Equation (1) into

(5) $$\ln\left(1-\frac{\sigma }{\left(A+B\varepsilon^{n}\right)\left(1+C\ln{\dot{\varepsilon }}^{*}\right)}\right)=m\ln T^{*}$$

where *m* is the slope of the linear fitting line in Figure 6, that is, 0.617.

All parameters in the constitutive model have been determined. The corresponding parameters are substituted into Equation (1), and the constitutive model of TA31 alloy can be obtained as

(6) $$\sigma =\left(893+956\varepsilon^{0.523}\right)\left(1+0.0098\ln\frac{\dot{\varepsilon }}{0.001}\right)\left(1-{\left(\frac{T-293}{1630}\right)}^{0.617}\right)$$

**Figure 7:**
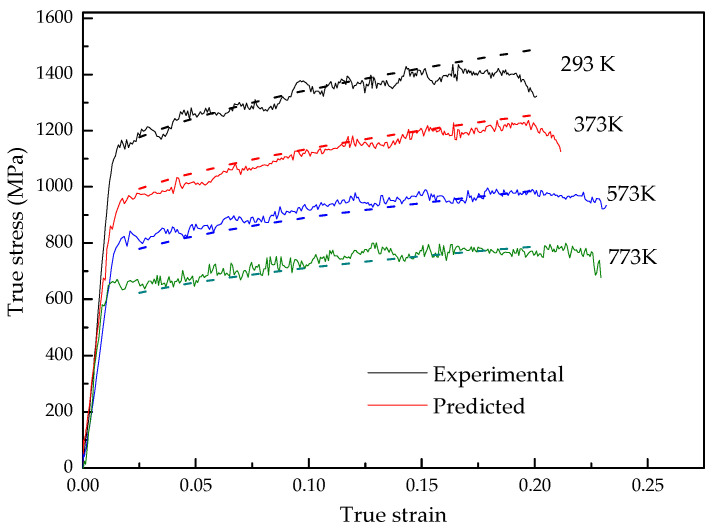
Comparison between the experimental and predicted flow stress at a strain rate of 1600 s^−1^ and deformation temperatures from 293 K to 773 K.

**Figure 8:**
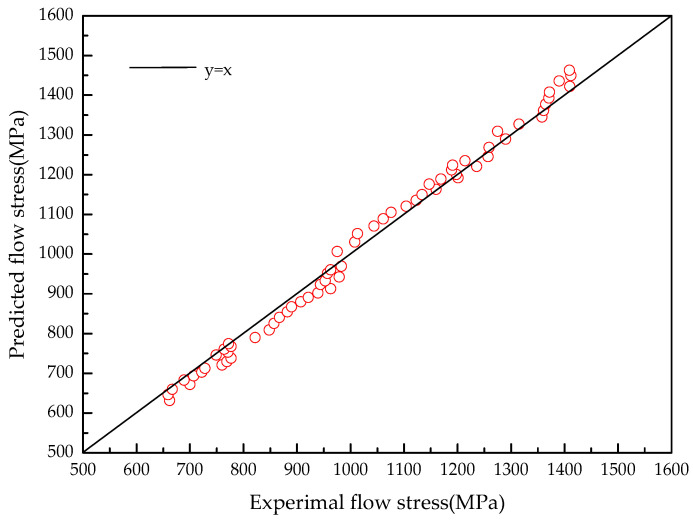
Correlation between the experimental and predicted flow stress by the model.

The prediction accuracy of the model is verified at deformation temperatures from 293 K to 773 K and strain rates from 0.001 s^−1^ to 4000 s^−1^. Figure 7 shows the comparison between the calculated results of the flow stress model established in this paper and the experimental data, and the relation between the experimental and predicted values are shown in Figure 8. It can be seen from Figure 7 and Figure 8 that the flow stress calculated by the flow stress model can achieve satisfactory accuracy. The average relative error between the predicted values by the model and the experimental data is 2.32%. Therefore, the flow stress model established in this paper can accurately describe the flow behavior of TA31 alloy during dynamic impact deformation.

### 3.3. Microstructure Evolution

**Figure 9:**
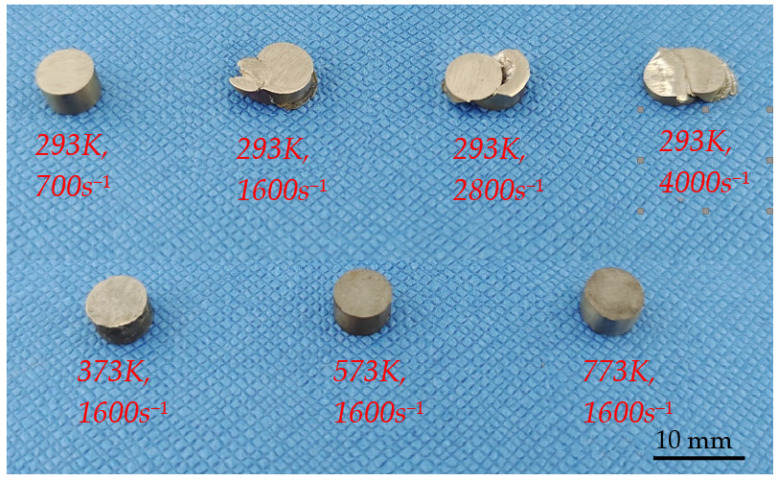
Photos of specimens deformed at different strain rates and temperatures.

Photos of the deformed specimens under dynamic impact load are shown in Figure 9. As shown in Figure 9, when the deformation temperature is 293 K and the strain rate is 700 s^−1^, there is no shear failure in the specimen after deformation, but when the strain rate is increased to 1600 s^−1^ or more, the specimen appears shear fracture. At the same strain rate of 1600 s^−1^, when the deformation temperature is 373 K, 573 K, and 773 K, there is no shear failure in the deformed specimens, which indicates that the increase of deformation temperature can improve the plasticity of TA31 alloy under dynamic impact condition.

**Figure 10:**
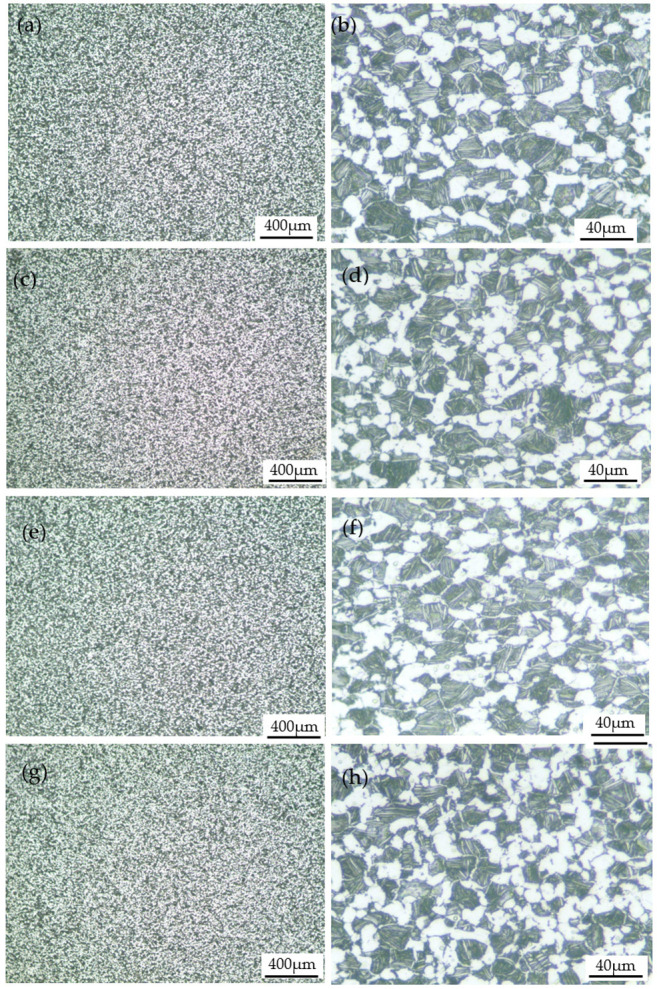
Optical images of specimens deformed at different temperatures and strain rates: (**a**,**b**) 293 K, 700 s^−1^; (**c**,**d**) 373 K, 1600 s^−1^; (**e**,**f**) 573 K, 1600 s^−1^; (**g**,**h**) 773 K, 1600 s^−1^.

Low and high-magnification optical microscope images of the deformed specimens without shear fracture are shown in Figure 10. It can be seen from the low magnification images, as shown in Figure 10a,c,e,g, that the microstructure of the deformed specimens is uniform, which indicates that there is no obvious inhomogeneous deformation. From the high magnification images, as shown in Figure 10b,d,f,h, it can be seen that the microstructure is composed of equiaxed primary α phase, secondary lamellar α phase, and β matrix phase. Compared with Figure 1, it can be seen that the microstructures of the deformed specimens have no obvious change under the corresponding impact deformation conditions. This is mainly due to the small degree of deformation (the true strain is about 0.2), and at the same time, the highest deformation temperature is only 773 K, which has no obvious effect on the dissolution and re-precipitation of the lamellar α phase.

**Figure 11:**
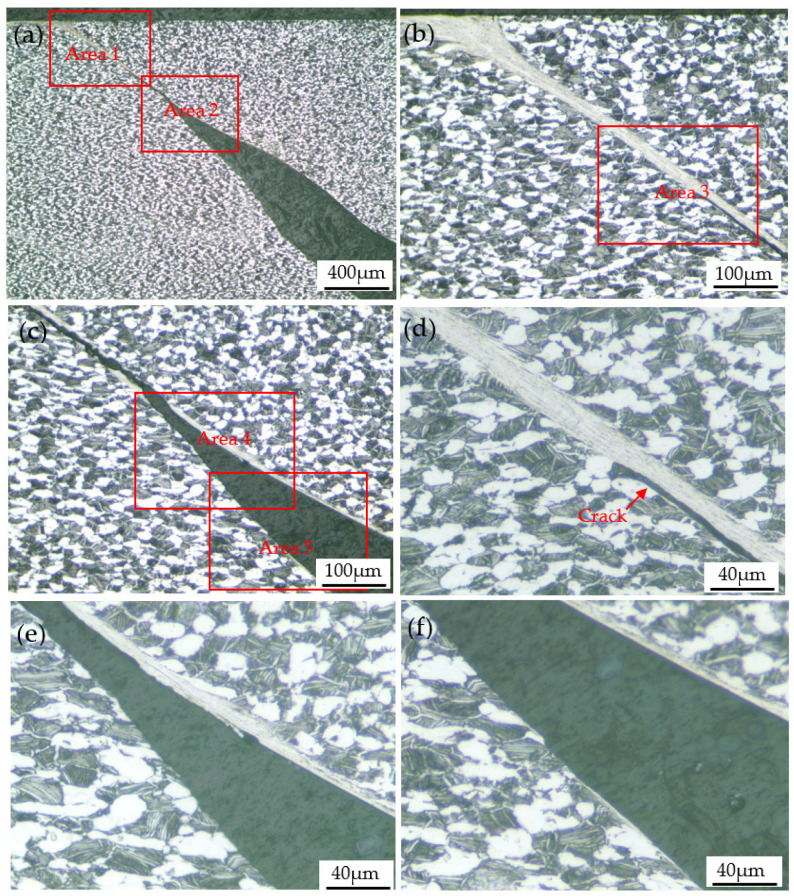
(**a**) Optical image of the specimen deformed at 293 K and 1600 s^−1^; (**b**) enlarged view of area 1 in (**a**); (**c**) enlarged view of area 2 in (**a**); (**d**) enlarged view of area 3 in (**b**); (**e**) enlarged view of area 4 in (**c**); (**f**) enlarged view of area 5 in (**c**).

Optical images of the specimen deformed at 293 K and 1600 s^−1^ are shown in Figure 11. As shown in Figure 11a, there is a crack inside the deformed specimen. A magnified view of the crack tip revealed a heavily deformed band-like region known as a shear band. The shear band is approximately 45° to the axial direction of the specimen, which indicates that the shear band is caused by shear stress. It can be seen from Figure 11d that the width of the shear band is about 20 μm, and the α phase inside the shear band is fibrous.

**Figure 12:**
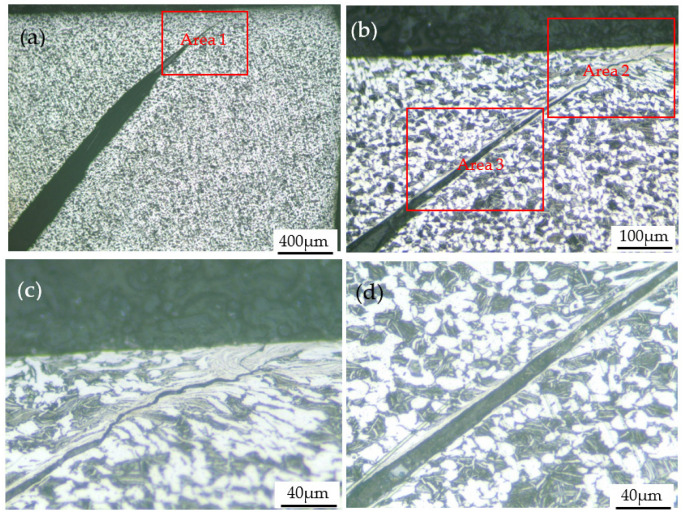
(**a**) Optical image of the specimen deformed at 293 K and 3200 s^−1^; (**b**) enlarged view of area 1 in (**a**); (**c**) enlarged view of area 2 in (**b**); (**d**) enlarged view of area 3 in (**b**).

**Figure 13:**
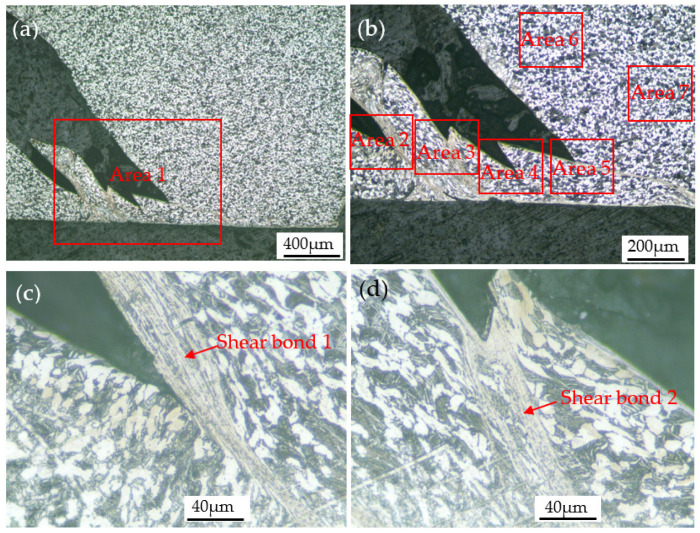

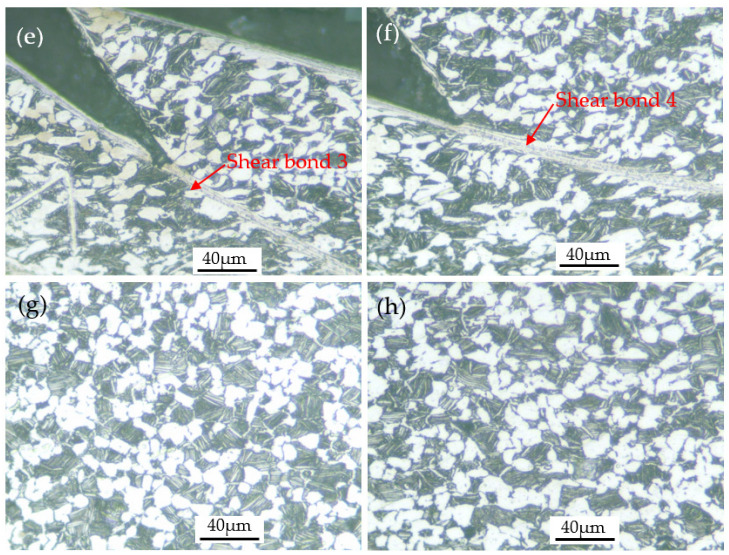
(**a**) Optical image of the specimen deformed at 293 K and 4400 s^−1^; (**b**) enlarged view of area 1 in (**a**); (**c**) enlarged view of area 2 in (**b**); (**d**) enlarged view of area 3 in (**b**); (**e**) enlarged view of area 4 in (**b**); (**f**) enlarged view of area 5 in (**b**); (**g**) enlarged view of area 6 in (**b**); (**h**) enlarged view of area 7 in (**b**).

When the deformation temperature was 293 K, and the strain rates were 2800 s^−1^ and 4000 s^−1^, shear bands similar to that in Figure 11 also appeared in the specimen and caused the specimen to fracture, as shown in Figure 12 and Figure 13, respectively. In particular, when the strain rate increased to 4000 s^−1^, four shear bands appeared inside the deformed specimen. The increase in the number of shear bands may be due to the fact that more shear bands can absorb the large energy input instantaneously under faster impact deformation conditions.

## 4. Discussion

### 4.1. Deformation Mechanism in Shear Bond

**Figure 14:**
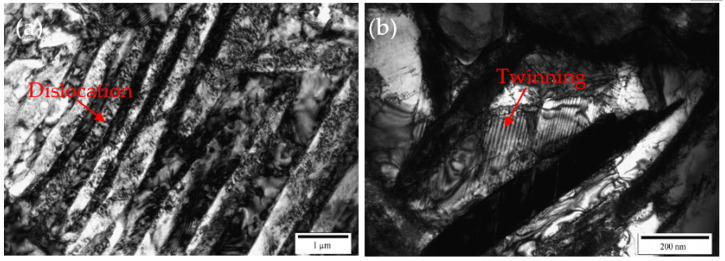
Transmission electron microscopy images of the shear bond in the specimen deformed at 293 K and 1600 s^−1^: (**a**) low magnification and (**b**) high magnification.

During the impact deformation process of titanium alloys, shear bands can often be observed. The microstructure characteristics of shear localization caused by flow instability of near α Ti-6Al-2Zr-1Mo-1V titanium alloy under impact load were studied, and it was found that two factors, geometry and structure, affected the nucleation and propagation of shear band [16]. Yang et al. [17] studied the evolution of shear localization in commercially pure titanium and found that a shear band with a width of about 25 μm was formed in the material under the impact load, and fine nano-grains with an average size of about 70 nm were observed in the center of the shear band. In the shear band of the Ti-5Al-2.5Cr-0.5Fe-4.5Mo-1Sn-2Zr-3Zn alloy, severely elongated grains in the direction parallel to the boundary of the shear band were found, and the width of the elongated grains is on the order of μm [18]. Jiang et al. [19] studied the microstructure characteristics of annealed pure titanium after dynamic compression and found that the microstructure in the shear band was significantly smaller than that of the surrounding metal, and the grain was elongated along the shear direction. Pavlenko et al. [20] studied the deformation mechanism of VT1-00 titanium alloy under impact load and found a high density of dislocations and twins, which indicated that in addition to dislocation slip, twining was often one of the deformation mechanisms during dynamic impact deformation. The research results of references [21,22,23,24] also show that the shear bands formed by high-speed impact deformation of titanium alloys are usually accompanied by high-density dislocations and twins. Similar findings have been made in the present study. It can be seen from Figure 11, Figure 12 and Figure 13 that the microstructure of the shear bonds is much finer than the surrounding material, and the α phase in the shear bonds is elongated into a fibrous shape along the deformation direction. As shown in the transmission electron microscope images in Figure 14, the width of the fibrous α phase in the shear band is about 200~500nm, and there are a large number of dislocations and twins in the α phase. This is because dislocation slip plays a leading role, and twinning plays a coordinating role in the deformation of titanium alloys, which indicates that the α phase in the shear band has undergone large shear deformation and has been significantly refined.

Recrystallization is usually observed in titanium alloy shear bands. Liu et al. [6] When studying the microstructure evolution of shear bands in Ti-6Al-4 V alloy during dynamic impact deformation, it was found that when the deformation temperature was 650 °C, complete recrystallization occurred, forming equiaxed grains, but when the deformation temperature was 25 °C, partial recrystallization occurred, forming a mixed structure of equiaxed and lathed grains. Highly elongated sub-grains and fine equiaxed grains were observed in the shear band on the titanium side of the interface of the explosive cladding of titanium/ mild steel, and the existence of fine equiaxed grains indicated that dynamic recrystallization occurred in the shear band [25]. The existence of shear bands was also observed during the dynamic impact deformation of the metastable β-titanium alloy Ti-6Mo-3.5Cr-1Zr, and the average grain size in the shear bands was found to be refined under the action of dynamic recrystallization [26]. A shear band is formed in TC17 titanium alloy during dynamic impact deformation, and it is found that dynamic recrystallization occurs in the β phase at the center of the shear band [27]. However, in this study, elongated fibrous grains and a high concentration of dislocations were present in the shear bands, as shown in Figure 14, and no equiaxed recrystallized grains were observed, suggesting that no obvious recrystallization occurred in the shear band. This may be due to the fact that the shear band was formed during impact deformation at room temperature; the deformation temperature was low and did not reach the critical recrystallization temperature.

The inside of the shear band has experienced a large plastic deformation and obtained significant work hardening, while the metal matrix outside the shear band is less deformed and has not been work hardened (the region outside the shear band maintains a microstructure similar to that of the as-received condition, i.e., equiaxed primary alpha phase, randomly distributed lamellar secondary alpha phase and beta matrix phase, as shown in Figure 13g,h, which led to a significant difference in metal strength and hardness between the two sides of the shear band boundary. This is also confirmed by the microhardness test results. The microhardness inside and outside the shear band is 370 HV and 315 HV, respectively, which indicates that the metal inside the shear band has been significantly strengthened. The strengthening mechanism includes work hardening (increase in dislocation density) and fine-grain strengthening. Similar findings were also found in the literature [9,17,19].

As shown in Figure 13, the angle between the shear band 1 and the axial direction of the specimen is close to 45°, which results in a larger width of the shear band, about 20–40 mm. The angle between the shear band 4 and the axial direction of the specimen is far from 45°, and the width of the shear band is small, about 10–15 mm. That is, the shear band near the direction of the maximum shear stress is wider. This rule can also be found in the shear band 2 and the shear band 3. This phenomenon is caused by the significant difference in strength and hardness inside and outside the shear band. In the impact deformation process, due to the high dislocation density inside the shear band, it is difficult for dislocations to move inside or cross the shear band, and the instantaneous plastic deformation is mainly concentrated at the edge of the shear band. Therefore, the continuous plastic deformation leads to the shear band gradually widening.

### 4.2. Relationship between Shear Bond and Fracture

Shear bands play an important role in the fracture process. It was found during the dynamic compression of titanium alloy-Ti17 alloy that the fracture of the specimen often occurred in the shear band [7]. Xue et al. [28] studied the evolution of the adiabatic shear band during the impact deformation of commercially pure titanium and Ti-6Al-4V alloy and found that the shear band is the first choice for nucleation, growth, and coalescence of voids, so it is the precursor of failure. The microstructure analysis of the adiabatic shear band of Ti-5Al-2.5Cr-0.5Fe-4.5Mo-1SN-2Zr-3Zn alloy shows that the crack is caused by the deformation incompatibility between the first recrystallization zone and the surrounding high work hardening zone [17]. Wei et al. [29] studied the fracture behavior of Ti-6Al-4V alloy under impact load and found that it is easier for the crack to propagate along the shear band than through the shear band because the lamellar α in the shear band hinders the crack propagation. It is found in the literature that the shear band is the channel for crack propagation, which shows that the shear band has an important influence on the fracture of the material. However, the exact location of the crack in the shear band was not clearly observed.

In this study, crack propagation along the shear band boundary can be clearly observed, as shown in Figure 11d–f. As analyzed in Section 4.1, during impact deformation, the instantaneous plastic deformation is mainly concentrated at the edge of the shear band. Because the α phase inside the shear band has undergone plastic deformation and is fibrous, and there are defects such as a high density of dislocations inside, which leads to a greater resistance for dislocations to pass through the fibrous α phase, dislocations mainly slip along the shear band boundaries. When the dislocation accumulates to a certain extent, or there are microcracks or metallurgical defects in the shear band boundaries, cracks will be generated. After the crack initiation, the stress concentration formed at the crack tip will cause the crack to expand until the material breaks. The crack propagation direction is the direction of the maximum shear stress, that is, at an angle of 45° to the external force.

## 5. Conclusions

In this work, the quasi-static and dynamic impact compression tests of the TA31 titanium alloy were conducted at the deformation temperatures from 293 K to 773 K and strain rates from 0.001 s^−1^ to 4000 s^−1^, and the dynamic compressive behavior, microstructure evolution, and fracture process were investigated. The following main results are obtained.

(1) the TA31 titanium alloy shows typical elastic-plastic characteristics. In the initial stage of compression (elastic stage), the flow stress increment is proportional to the strain increment, and the stress–strain curve is a straight line. In the plastic deformation stage, deformation temperature has a significant effect on the flow stress, and the flow stress decreases with increasing deformation temperature. The effect of strain rate on flow stress during dynamic compression is insignificant.

(2) The J-C constitutive model of TA31 alloy is established and verified at deformation temperatures from 293 K to 773 K and strain rates from 0.001 s^−1^ to 4000 s^−1^, and the average relative error is 2.32%, which can accurately describe the deformation behavior of TA31 titanium alloy under dynamic impact loading.

(3) When the deformation temperature is 273 °C, and the strain rate reaches 1600 s^−1^, a shear band with an angle of about 45° to the axial direction of the specimen appears. The plastic deformation is mainly concentrated in the shear band. The severe shear deformation makes the α phase in the shear band fibrous and contains high-density dislocations. Increasing the strain rate can promote the generation of shear bands. When the strain rate reaches 4000 s^−1^, four shear bands appear inside the sample. However, increasing the deformation temperature will inhibit the generation of shear bands.

(4) The instantaneous plastic deformation during the impact deformation process is mainly concentrated on the edge of the shear band, and the continuous plastic deformation makes the shear band gradually widen.

(5) During the shear fracture process, the crack propagates along the boundary of the shear band, which eventually leads to the fracture of the specimen.

## Figures and Tables

**Table 1:** Chemical composition of the TA31 titanium alloy (in wt.%).

Ti	Al	Zr	Mo	Nb	C	N	H	O
Bal.	6.13	2.06	0.97	2.96	≤0.01	≤0.005	≤0.001	≤0.09

## Data Availability

The data presented in this study are available on request from the corresponding author.
